# JTM’s Tumor immunology goes broad: announcing the Immunobiology and Immunotherapy section

**DOI:** 10.1186/1479-5876-11-2

**Published:** 2013-01-04

**Authors:** Adrian Bot, Francesco Marincola, Pedro Romero

**Affiliations:** 1 Kite Pharma, Inc, 10924 Le Conte Avenue, Los Angeles, CA 90024, USA; 2 Incoming Section Editor of Immunobiology and Immunotherapy of JTM, Bethesda, USA; 3 Infectious Disease and Immunogenetics Section (IDIS), Department of Transfusion Medicine, Clinical Center, Bethesda, MD, USA; 4 Trans-NIH Center for Human Immunology, National Institutes of Health, Bethesda, MD, USA; 5 Editor in Chief of JTM, Bethesda, USA; 6 Ludwig Center for Cancer Research of the University of Lausanne, Avenue Pierre-Decker 4, Lausanne, 1011, Switzerland; 7 Incoming Editor in Chief of the Journal for Immunotherapy of Cancer, the new BMC journal of the Society for Immunotherapy of Cancer (SITC), Lausanne, Switzerland

## Abstract

For the last four years the Journal of Translational Medicine (JTM) has hosted the Section of Tumor Immunology and Biological Cancer Therapy. Under the editorial leadership of Dr. Pedro Romero and with the direct support of the Society for Immunotherapy of Cancer (SITC), this section enriched the communication between basic immunological sciences and the clinical investigation arena in oncology. We are re-launching this Section of JTM, now entitled Immunobiology and Immunotherapy, succeeding Tumor Immunology and Biological Cancer Therapy. While aiming to build on the editorial success and focus of its predecessor, this novel Section will have a broader scope, hosting translational immunology topics pertaining to immunotherapy beyond oncology, including disciplines such as inflammation, autoimmunity, transplantation, metabolic disorders and others. As the vision of this re-launched Section of JTM broadens up to serve a communication need for translational immunologists involved with immunotherapy irrespectively of the therapeutic area, a novel and focused journal entitled Journal for Immunotherapy of Cancer (JITC) has just been initiated, sponsored by the SITC.

## 

During the last four years the JTM’s Section of Tumor Immunology and Biological Cancer Therapy, under the editorial leadership of Dr. Pedro Romero and with the direct support of the Society for Immunotherapy of Cancer (SITC) [1], has hosted nearly 70 articles – editorials, reviews, research articles and others – in accordance to its mission to enrich the communication between basic immunological scientists and the clinical investigators in oncology. The vision and objectives of this section co-sponsored by the Society for Immunotherapy of Cancer SITC (formerly the International Society for Biological Therapy of Cancer or iSBTc), together with BioMed Central, were articulated in an Editorial by Romero, Fox and collaborators [2]. With that step, JTM became a venue for publication of original research articles, literature reviews, opinion/position papers and a forum to discuss the hot issues in tumor immunology highly relevant to translating novel immune interventions. This Section came at a turning point in cancer immunotherapy, when considerable advances in understanding the relationship between the immune system and cancer started to translate to pioneering active immunotherapies such as the dendritic cell vaccine Provenge® and the checkpoint blocking antibody Ipilimumab® now approved for clinical use [3,4]. But this was just the start as this scientific progress in oncoimmunology fueled unprecedented interest and activity in designing and translating novel immunotherapies, standalone or in context of rationally selected combinatorial approaches. Paralleling these exciting developments in oncoimmunology, a novel journal on its own right, sponsored by SITC, is taking on the mission to provide a venue for the ever growing scientific publication and communication need in this very field. Journal for Immunotherapy of Cancer (JITC) [5] an open access, peer reviewed journal, will accept various types of submissions in basic tumor immunology, clinical and translational cancer immunotherapy, and immune biomarkers with prognostic or predictive value. In addition, the new journal will host comments and discussion on timely topics and innovative concepts. JITC thus aims to create a dynamic and global platform for interaction and discussion on topics spanning science, policy, strategy and implementation of cancer immunotherapy.

We are now also re-launching this section of JTM, to be entitled Immunobiology and Immunotherapy, succeeding the Tumor Immunology and Biological Cancer Therapy section. Through this step, the mission of JTM will expand with this redesigned section with a much broader scope but outside the purview of the SITC. This renewed section will host translational immunology topics pertaining to immunotherapy beyond oncology, including disciplines such as inflammation, autoimmunity, transplantation, metabolic disorders and others. As immunology has evolved to be a central discipline connected to a wide range of therapeutic areas, the number and diversity of platform technologies also increased, spanning small molecules, biologics, microbial vectors and cells. As the vision of this novel section of JTM broadens up to serve a communication need for translational immunologists involved with immunotherapy irrespectively of the therapeutic area, a cross fertilization between various fields such as autoimmunity, infection and cancer, could dramatically catalyze the process of discovery and translation of innovative immunotherapies. To illustrate that principle, it suffices to refer to the definition of the “immunological constant of tissue rejection” [6], a gene signature that connects immune mediated processes across the board and that could serve as a rich source of druggable targets and companion biomarkers for novel immune interventions designed to either amplify or quench immune reactions in widely different therapeutic areas. This expansion in the scope of JTM effected by the introduction of this new section of Immunobiology and Immunotherapy mirrors a recent addition to the journal, namely the section of Patient-Targeted Molecular Therapies [7], that provides a venue for targeted therapies and translational effectiveness pertaining to diverse therapeutic areas including inflammation, degenerative disorders, cancer, infection, metabolism, neurologic, cardiovascular and genetic diseases, irrespectively of the therapeutic strategy.

The Section of Immunobiology and Immunotherapy will host a broad range of submissions in accordance with JTM’s mission [8]: research articles, commentaries, meeting reports, methodologies, protocols and reviews.

On behalf of the JTM we are launching the invitation to submit your work and provide your suggestions for the Section of Immunobiology and Immunotherapy. We firmly believe that our journal’s commitment to provide rapid publication, open-access and online communication at global level is key to our vision to become and remain the premier translational medicine journal worldwide. At last but not least, we are very thankful to the Editorial team of the previous Section of Tumor Immunology and Biological Cancer Therapy and we invite them to continue to support JTM as we are expanding the team with experts across diverse therapeutic areas.
